# Necrotizing Fasciitis as a Novel Complication of Penile Edema in a Patient With Monkeypox: A Case Report

**DOI:** 10.7759/cureus.78509

**Published:** 2025-02-04

**Authors:** Sara S Gawargeous, Aysha M Almeqbaali, Omar A Al Hammadi

**Affiliations:** 1 Internal Medicine, Al Rahba Hospital, Abu Dhabi, ARE

**Keywords:** infectious disease medicine, male circumcision, monkeypox virus, necrotizing fasciitis, penile edema

## Abstract

Unlike classical smallpox and other orthopoxviruses, primary genital lesions may be the initial presentation of monkeypox, particularly in sexually active or immunocompromised individuals, with a rising incidence in certain populations. We report a case of monkeypox with an unusual presentation of penile edema and phimosis in an uncircumcised male complicated by bacterial superinfection and necrotizing fasciitis. The patient presented with fever, painful genital swelling, and a vesiculopapular rash that progressed to involve the peripheries. Prompt diagnosis and surgical intervention, including circumcision, debridement, and appropriate antibiotic coverage, effectively managed the necrotizing fasciitis and improved the patient’s outcome. This case highlights the need for heightened clinical suspicion of monkeypox in patients presenting with genital lesions, as early diagnosis and management are critical in preventing severe complications.

## Introduction

Monkeypox belongs to the *Orthopoxvirus* genus and resembles smallpox in the presentation of skin rash, making it challenging to differentiate clinically from smallpox or chickenpox [1,2]. In May 2022, the World Health Organization declared a monkeypox outbreak, and by July 2022, it was classified as a global health emergency, with more than 16,000 cases reported across 75 non-endemic countries [3,4]. Monkeypox is a zoonotic infectious disease recently recognized to be transmitted between humans through various routes, including close physical contact with lesion exudates and infected body fluids, such as saliva or respiratory droplets [5,6]. A study conducted in the United Kingdom on confirmed monkeypox patients from May to early July 2022 reported that 78.8% of cases occurred among individuals with bisexual, homosexual, or heterosexual contacts, indicating a novel transmission route in non-endemic countries [5]. All patients presented with mucocutaneous lesions, most commonly on the genitals or perianal area, with or without systemic illness [7].

Monkeypox has an incubation period of around one to two weeks, followed by a flu-like prodromal illness. Monkeypox disease presents with localized lymphadenopathy, which occurs in various body parts, even when the disease is generalized. This characteristic differentiates it from smallpox in presentation. The rash progresses through four stages: macular, papular, vesicular, and pustular, eventually scabbing over and resolving. The disease course typically lasts two to four weeks. Lesions are initially painful and later become itchy. The patient is no longer contagious once the lesions have crusted and fallen off. The clinical presentation and severity vary among patients, depending on general health, age, and comorbidities, with immunocompromised individuals experiencing more severe manifestations [8].

Penile edema and rectal pain have been identified as new clinical presentations in sexually active patients. A high index of suspicion for monkeypox should be maintained in such cases, as early diagnosis and management can help reduce onward transmission [9,10].

When clinically indicated, monkeypox viral polymerase chain reaction (PCR) is the preferred laboratory test due to its high accuracy and sensitivity. Samples are collected from both dry and wet lesions. Additionally, throat and urine swabs are commonly taken as part of the criteria for discharging patients back into the community [7].

Brincidofovir and tecovirimat are currently undergoing human trials to evaluate their efficacy in the treatment of monkeypox. Both drugs have demonstrated efficacy against orthopoxviruses, including monkeypox, in animal models. JYNNEOS and ACAM2000 are two vaccines now available that may be used to prevent monkeypox virus infection for high-risk individuals, either post-exposure prophylaxis or pre-exposure prophylaxis [2,11].

## Case presentation

**Figure 1:**
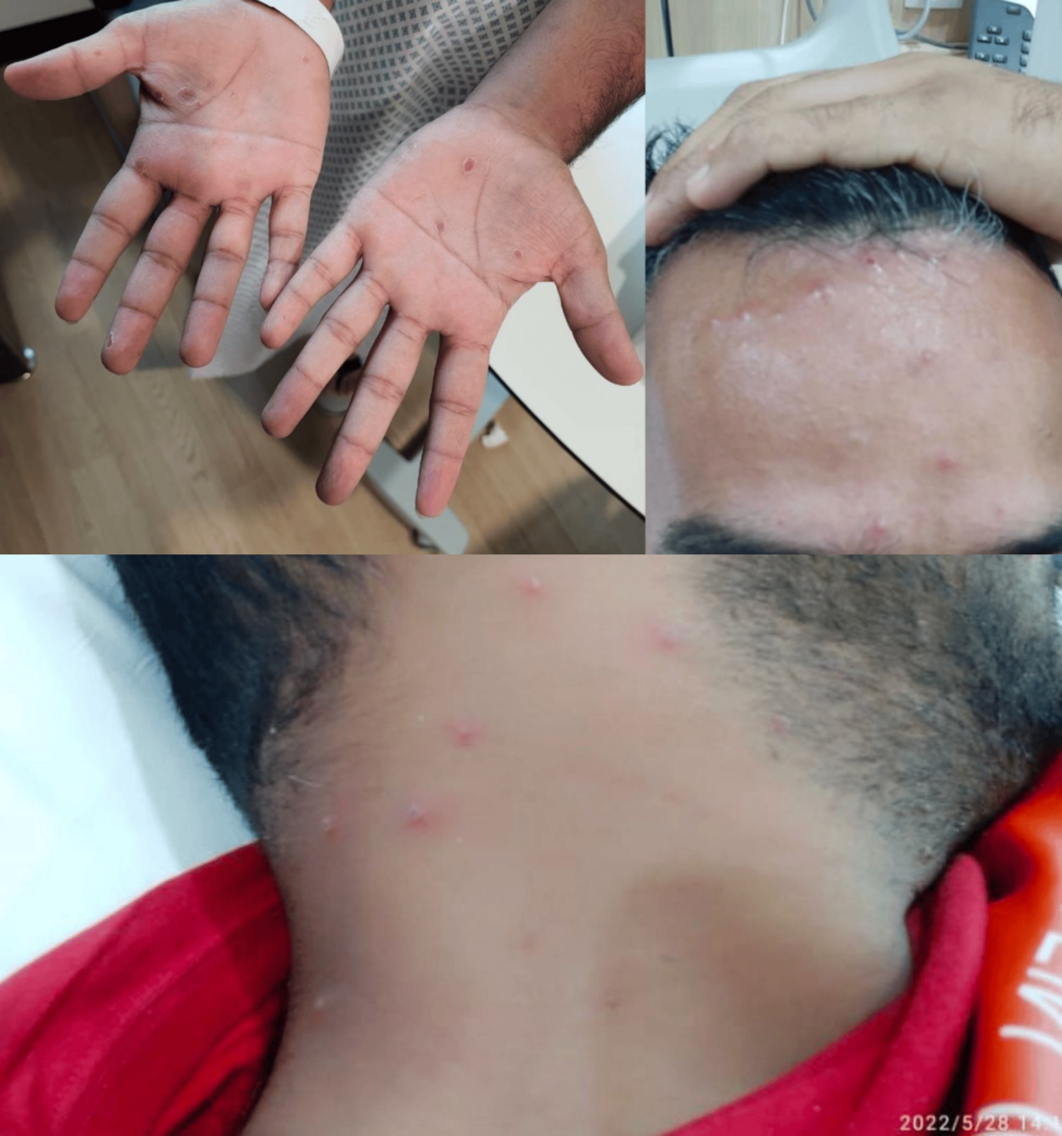
Maculopapular, vesicular, and pustular lesions on the face, neck, and palms

A 25-year-old uncircumcised male presented with a four-day history of fever, followed by the development of a vesiculopapular rash on the penis and scrotum, accompanied by left-sided painful inguinal swelling. Over the next few days, the rash spread to the face, palms, and soles (Figure 1). The patient also reported pain and difficulty urinating. He denied recent travel, but he had unprotected sexual contact with a female partner one month prior to the presentation. He denied sexual contact with males, although social barriers made obtaining a full sexual history challenging. The patient works as a housekeeper in a hotel. He denies smoking or using illicit drugs but drinks alcohol occasionally. Additional history revealed chickenpox during childhood, though he has no history of varicella zoster vaccination.

Hospital course

The patient was admitted as a suspected case of monkeypox. Investigations were conducted, including a complete blood count, renal function tests, liver function tests, and screening for sexually transmitted diseases (STDs). Additionally, monkeypox PCR was obtained from both wet and dry lesions, and an ultrasound of the inguinal swelling was requested. The complete blood profile, including renal function and white blood cell count, was within normal limits, except for a markedly high C-reactive protein level of 136 mg/dL, indicating acute inflammation. The inguinal ultrasound showed a fluid collection measuring 3.7 x 1.2 x 2.6 cm in the left inguinal region, with multiple lymph nodes observed on both sides, all with retained fatty hilum, likely reactive.

**Figure 2:**
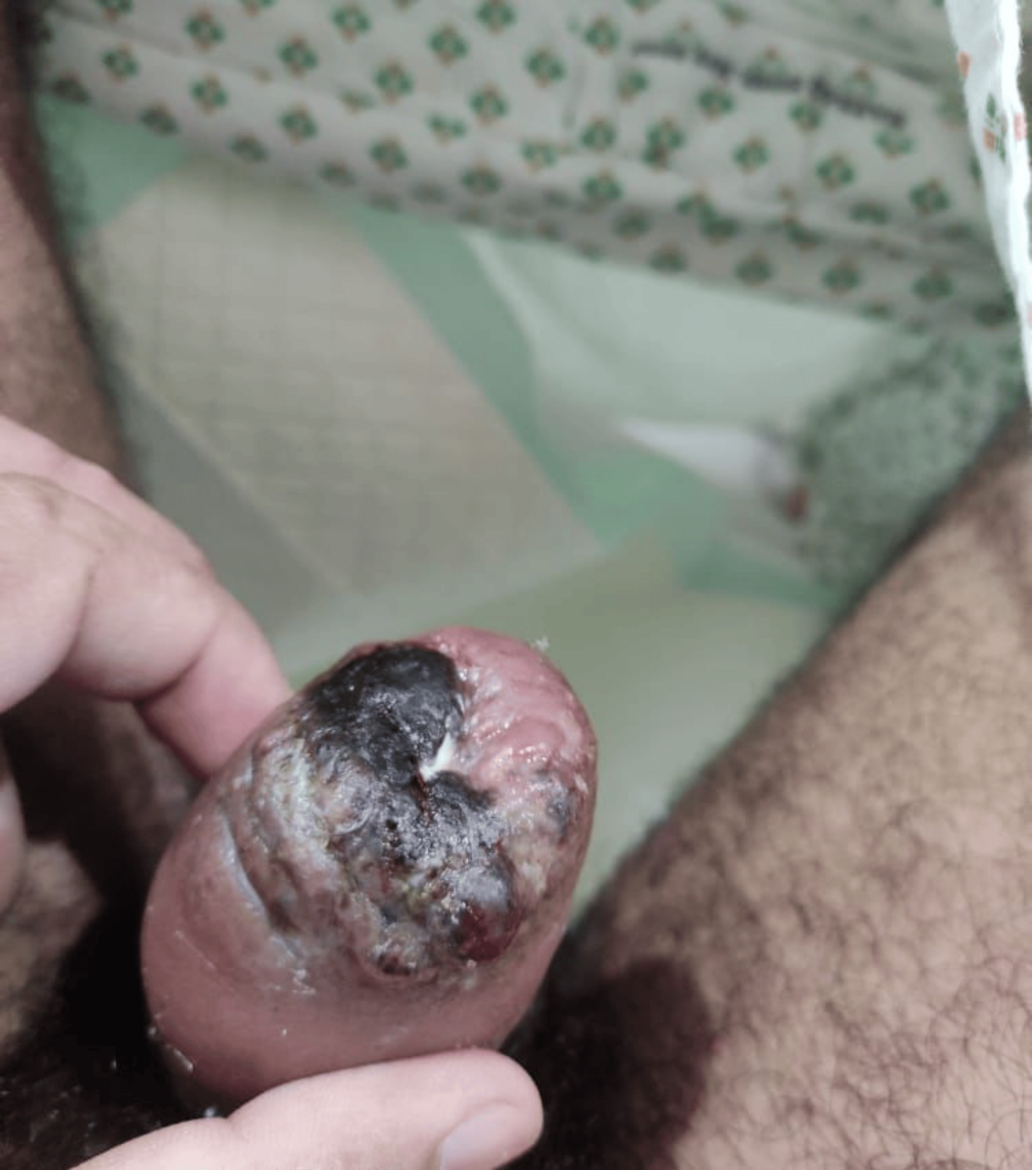
Genital examination showing moderate penile swelling and blackish discoloration of the distal foreskin

The following day, the patient remained stable but reported severe penile pain. On examination, a vesiculopapular rash was noted on the forehead, arms, and soles. Genital examination revealed moderate penile swelling, blackish discoloration of the distal foreskin, and an inability to retract it, along with a tender left inguinal swelling. Acute phimosis with necrosis was clinically suspected. The patient was started on intravenous meropenem and vancomycin empirically. An urgent urology review was conducted, which revealed blackish necrotic foreskin with pus and the inability to retract the foreskin to expose the glans, with the impression of early necrotizing fasciitis (Figure 2).

**Figure 3:**
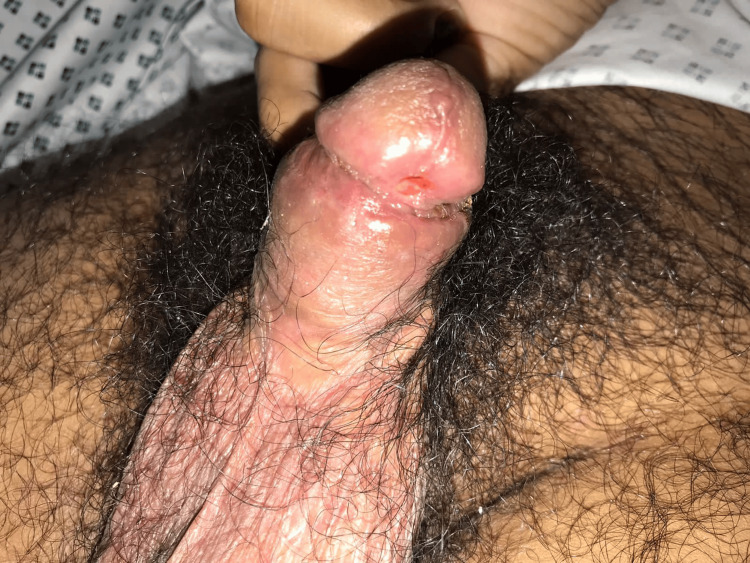
Surgical circumcision of the foreskin showing an underlying penile ulcer

The patient underwent urgent circumcision and debridement on the same day. The procedure was uneventful, and the necrotic tissue was sent for histopathology and culture. Removal of the foreskin revealed an underlying penile ulcer (Figure 3). Monkeypox PCR was positive, and STD screening revealed *Neisseria gonorrhoeae* and *Chlamydia trachomatis*. HIV, hepatitis B, and C screening was negative. The patient completed five days of meropenem and vancomycin, and based on infectious disease recommendations, the patient was switched to intravenous piperacillin-tazobactam and doxycycline for seven additional days.

Follow-up and outcome

Over the next few days, the patient showed clinical improvement. The body rash crusted over, revealing new underlying skin, and penile edema and pain resolved. Histopathology of the circumcised foreskin revealed extensive severe acute suppurative inflammation, skin ulceration, subcutaneous tissue necrosis, subcutaneous vessel thrombosis, and bacterial colonization. The histopathologic findings were suggestive of early necrotizing fasciitis. However, the surgical skin and subcutaneous margins were viable, showing only mild acute inflammation.

Despite improvement in other areas, the penile ulcer persisted and failed to heal for a few weeks. Urology was re-consulted for the non-healing ulcer, initially thought to be related to the circumcision. A repeat monkeypox PCR from the penile ulcer confirmed that the lesion was still a monkeypox lesion rather than a post-procedure ulcer. After five weeks, the monkeypox PCR result was negative, and all skin lesions had healed with new skin formation. The genital lesion also healed, becoming indistinguishable from normal skin.

The patient was discharged to the community after receiving a negative monkeypox PCR from the throat and a resolved skin rash. Follow-up was scheduled with both urology and the infectious disease clinic.

## Discussion

This case underscores the emerging complications of monkeypox, particularly in sexually active patients [1]. Penile edema and phimosis, previously underreported, are now recognized as potential complications that affect uncircumcised men with monkeypox [2]. Genital involvement in sexually active individuals highlights the need to consider monkeypox in patients with genital lesions, especially with inguinal lymphadenopathy and systemic symptoms [3].

In this case, the patient’s progression to necrotizing fasciitis emphasizes the importance of early intervention. Surgical management, including circumcision and debridement, was essential to prevent further complications [4]. Antibiotic therapy, guided by microbiological findings, was pivotal in managing secondary infections, including gonorrhea and chlamydia [5]. This case highlights the diagnostic challenges of monkeypox in the context of mixed infections and underscores the importance of comprehensive diagnostic workups [6].

Although monkeypox typically resolves with supportive care, complications such as necrotizing fasciitis, particularly in immunocompromised or sexually active patients, may result in severe outcomes [7]. Early diagnosis, appropriate antimicrobial therapy, and timely surgical intervention significantly improve prognosis. Vaccines like JYNNEOS and antivirals such as tecovirimat offer promising prevention and treatment options, especially for high-risk individuals [8,9].

## Conclusions

With the outbreak of monkeypox, several novel clinical presentations have emerged, including penile edema, soft tissue swelling, and lymphadenitis. Penile edema with phimosis should be considered a potential complication of the monkeypox rash in the genitalia of uncircumcised men. This can progress to necrotizing fasciitis if not managed appropriately. A high index of suspicion is essential for diagnosing and managing these life-threatening complications, as early detection improves outcomes. However, prevention and limiting transmission remain key factors in disease control.
